# Age-Related Declines in Prefrontal Cortical Expression of Metabotropic Glutamate Receptors that Support Working Memory

**DOI:** 10.1523/ENEURO.0164-18.2018

**Published:** 2018-06-28

**Authors:** Caesar M. Hernandez, Joseph A. McQuail, Miranda R. Schwabe, Sara N. Burke, Barry Setlow, Jennifer L. Bizon

**Affiliations:** 1 Department of Neuroscience, University of Florida, Gainesville, FL 32610; 2 Department of Psychiatry, University of Florida, Gainesville, FL 32610

**Keywords:** aging, metabotropic glutamate receptor, prefrontal cortex, prelimbic cortex, rat, working memory

## Abstract

Glutamate signaling is essential for the persistent neural activity in prefrontal cortex (PFC) that enables working memory. Metabotropic glutamate receptors (mGluRs) are a diverse class of proteins that modulate excitatory neurotransmission via both presynaptic regulation of extracellular glutamate levels and postsynaptic modulation of ion channels on dendritic spines. This receptor class is of significant therapeutic interest for treatment of cognitive disorders associated with glutamate dysregulation. Working memory impairment and cortical hypoexcitability are both associated with advanced aging. Whether aging modifies PFC mGluR expression, and the extent to which any such alterations are regionally or subtype specific, however, is unknown. Moreover, it is unclear whether specific mGluRs in PFC are critical for working memory, and thus, whether altered mGluR expression in aging or disease is sufficient to play a causative role in working memory decline. Experiments in the current study first evaluated the effects of age on medial PFC (mPFC) mGluR expression using biochemical and molecular approaches in rats. Of the eight mGluRs examined, only mGluR5, mGluR3, and mGluR4 were significantly reduced in the aged PFC. The reductions in mGluR3 and mGluR5 (but not mGluR4) were observed in both mRNA and protein and were selectively localized to the prelimbic (PrL), but not infralimbic (IL), subregion of mPFC. Finally, pharmacological blockade of mGluR5 or mGluR2/3 using selective antagonists directed to PrL significantly impaired working memory without influencing non-mnemonic aspects of task performance. Together, these data implicate attenuated expression of PFC mGluR5 and mGluR3 in the impaired working memory associated with advanced ages.

## Significance Statement

Working memory is impaired in several neuropsychiatric disorders and advanced aging. Glutamate is essential for persistent cellular activity in the prefrontal cortex (PFC) theorized to maintain working memory. Metabotropic glutamate receptors (mGluRs) are well positioned to coordinate glutamate signaling at PFC synapses; however, studies to date have not yet systematically investigated the contributions of mGluR subtypes to normal working memory and PFC aging. This study shows that aging is accompanied by loss of PFC mGluR2/3 and mGluR5 mRNA and protein and that pharmacological inhibition of these mGluR subtypes is sufficient to impair working memory. These findings suggest that mGluRs have a normal role in working memory and could serve as a target for treatment of cognitive disorders characterized by PFC dysfunction.

## Introduction

Working memory involves the temporary representation of information to guide goal-directed behavior and is a foundational aspect of higher order cognition that is ascribed to the prefrontal cortex (PFC; Baddeley, 1986; Goldman-Rakic, 1996). The neural basis of working memory is theorized to depend on persistent firing of PFC pyramidal neurons that requires recurrent excitation of ionotropic glutamate receptors (Goldman-Rakic, 1995; Wang et al., 2013). Comparatively less work, however, has considered a role for slower, modulatory signaling achieved via metabotropic glutamate receptors (mGluRs). The mGluRs belong to the class C family of G protein-coupled receptors (Tanabe et al., 1992; Bjarnadóttir et al., 2006) and are subdivided into three groups on the basis of their sequence homology and downstream signaling mechanisms (Bishop and Ellingrod, 2007). In dendritic spines, Group I and some Group II mGluRs regulate ion channel activity and intracellular Ca^2+^ release to influence neural excitability (Mannaioni et al., 2001; Tyszkiewicz et al., 2004; Hagenston et al., 2008; Niswender and Conn, 2010; Arnsten et al., 2012; Jin et al., 2017). Also essential regulators of extracellular glutamate, Group II and III mGluRs localize to excitatory terminals and glial processes where they modulate the synaptic release of glutamate (Tanabe et al., 1993; Okamoto et al., 1994; Sansig et al., 2001) and glutamate uptake (Aronica et al., 2003; Corti et al., 2007), respectively.

The mGluRs are of significant therapeutic interest for treating PFC glutamate dysregulation and working memory dysfunction in several neuropsychiatric diseases, including schizophrenia and major depressive disorder. It is unclear whether deficient mGluR expression is causally linked to the working memory impairments observed in these conditions, however, as some studies show reductions in PFC Group I and Group II mGluR expression in schizophrenia and depression (Ghose et al., 2009; Corti et al., 2011; Deschwanden et al., 2011; McOmish et al., 2016), whereas others do not (Crook et al., 2002; Frank et al., 2011; Matosin et al., 2013). Aside from their potential roles in cognitive dysfunction in disease states, a secondary observation from this literature is that expression of at least some mGluR subtypes appears to decline across the lifespan, independent of the manifestation of psychiatric conditions (Crook et al., 2002; Corti et al., 2011; Frank et al., 2011). These initial observations suggest that attenuated mGluR expression with age may be a contributing factor to the precipitous working memory decline that often accompanies aging (Oscar-Berman and Bonner, 1985; Dunnett et al., 1988; Rapp and Amaral, 1989; Bachevalier et al., 1991; Lamar and Resnick, 2004; Beas et al., 2013; McQuail et al., 2016; Hernandez et al., 2017). Importantly, however, the effects of age on mGluR expression have only been examined in retrospective studies in populations with neuropsychiatric disease.

The overarching goal of the current study was to comprehensively evaluate mGluR expression in aged rat medial PFC (mPFC), the rodent homolog of primate dorsolateral PFC. The findings indicate that mGluR3 and mGluR5 expression decline specifically in the prelimbic (PrL) but not infralimbic (IL) subregion of mPFC. All other mGluRs were largely stable with age in both PFC subregions. Importantly, blockade of either mGluR2/3 or mGluR5 in the PrL reliably impaired working memory performance in young rats. Together, these data implicate selective reductions in PrL mGluR expression in age-associated working memory decline and suggest that targeting these receptors may have potential for improving working memory in aging and other disorders.

## Materials and Methods

### Subjects

Young adult (four months, *n* = 38) and aged (22 months, *n* = 30) male Fischer 344 (F344) rats were obtained from the National Institute on Aging’s Aging Rodent Colony maintained by Charles River Laboratories. All animals were housed in the Association for Assessment and Accreditation of Laboratory Animal Care International-accredited vivarium facility in the McKnight Brain Institute Building at the University of Florida. The facility was maintained at a consistent temperature of 25°C with a 12/12 h light/dark cycle (lights on at 7 A.M.) with free access to food and water except as otherwise noted. All animal procedures were reviewed and approved by the University of Florida Institutional Animal Care and Use Committee and followed National Institutes of Health guidelines. In experiment 1, a cohort of young adult (*n* = 8) and aged (*n* = 15) rats was used to measure protein expression of mGluR subtypes in the whole mPFC. Experiment 2 used a second cohort of young adult (*n* = 8) and aged (*n* = 15) rats to assess expression of gene transcripts that encode mGluR subtypes in the PrL and IL subregions of the mPFC. In experiment 3, young adult rats (*n* = 22) were used to probe the functional consequences of the age-related declines in mGluR expression identified in experiments 1 and 2, by evaluating the effects of pharmacological blockade of mGluR5 (*n* = 11) or mGluR2/3 (*n* = 11) in PrL on performance in a delayed response task used to assess working memory.

### Experiment 1: effect of age on expression of mGluR protein in the mPFC

#### Tissue dissection and protein extraction

Animals were killed by decapitation and the mPFC was micro-dissected from surrounding tissues on an ice-cold plate before freezing on dry ice and storage at -80°C until membranes were prepared (McQuail et al., 2012). All tissue samples were weighed and homogenized in 2-ml glass-Teflon dounce homogenizers containing ten volumes of 50 mM HEPES (pH 7.4) supplemented with 1 mM EDTA, 1 mM EGTA, and protease inhibitors (Halt from ThermoFisher). Tissue homogenates were transferred to a 1.5-ml tube, then centrifuged at 10,000 × *g* for 20 min at 4°C. The pellet, comprising the membrane-bound protein fraction, was resuspended in the same buffer and incubated on ice for 30 min. All samples were then centrifuged at 20,000 rpm (32,539 × *g*) for 10 min at 4°C. Finally, the washed pellet was resuspended in 50 mM HEPES buffer, then aliquoted and stored at -80°C until used for Western blot analyses.

#### SDS-PAGE and immunoblotting

Unless otherwise noted, all reagents used were from Bio-Rad. Each mPFC protein sample was diluted and reduced in Laemmli buffer with 5% (v/v) β-mercaptoethaol and denatured at 95°C for 5 min. A total of 5 μg of membrane protein was loaded per well in a 26 lane TGX 4–15% polyacrylamide gel. Each sample was assayed in duplicate and the location of each replicate was systematically varied between gels. Protein samples were separated for 45 min at 200 V in 1× running buffer (25 mM Tris, 192 mM glycine, and 0.1% SDS; pH 8.3). Resolved proteins were electrophoretically transferred to nitrocellulose membranes (0.45-μm pore size) in 1× transfer buffer (25 mM Tris and 192 mM glycine; pH 8.3) with 20% (v/v) methanol at 100 V for 30 min at 4°C. Membranes were then blocked in Rockland Blocking buffer for 1 h at room temperature. Proteins of interest were detected by overnight incubation with specific primary antibodies (Table 1) diluted in blocking buffer supplemented with 0.1% Tween 20 at 4°C. For each primary antibody, the optimal dilution was empirically determined to obtain a linear range of detection for 1.25–10 μg of mPFC membrane protein. Membranes were washed in 1× tris-buffered saline before incubation with IR-Dye conjugated secondary antibodies (Table 1). Excess secondary antibody was removed by washing with TBS + 0.1% Tween 20 (TBST) followed by additional washes of TBS. The membranes were then imaged on a LiCor Odyssey scanner and integrated intensity of immunoreactive bands was assessed using ImageStudio v3.2.

**Table 1.** Antibodies used for immunoblotting

Primary antibody	Made in	Supplier	Part number	Dilution	Secondary antibody (dilution)^*^
Anti-mGluR1	Rb	Millipore	07-617	1:500	Dk anti-Rb IRDye 700 (1:20,000)
Anti-mGluR5	Ms	Millipore	MABN540	1:500	Dk anti-Ms IRDye 800 (1:15,000)
Anti-mGluR2/3	Rb	Millipore	06-676	1:1000	Dk anti-Rb IRDye 700 (1:20,000)
Anti-mGluR4	Rb	Millipore	AB15097	1:1000	Dk anti-Rb IRDye 800 (1:15,000)
Anti-mGluR7	Gt	Abcam	ab85343	1:1000	Dk anti-Gt IRDye 700 (1:20,000)
Anti-mGluR8	Gt	Santa Cruz Biotechnology	sc-30300	1:500	Dk anti-Gt IRDye 800 (1:15,000)
Anti-α-tubulin	Ck	Sigma-Aldrich	SAB3500023	1:2000	Dk anti-Ck IRDye 700 (1:20,000)

Ck = chicken, Dk = donkey, Gt = goat, Ms = mouse, Rb = rabbit.

* All secondary antibodies were purchased from LI-COR Bioscience.

#### Statistical analysis of protein levels

Integrated intensities were normalized using α-tubulin as a loading control, which did not change with age in any of the individual experiments (*t*s = 0.238–0.397, *p*s = 0.695–0.814). Data were transformed to percentage level of young (i.e., mean level of young = 100%) and analyzed by independent-samples *t* test to compare protein levels between young and aged using the Benjamini–Hochberg method to correct for multiple comparisons with a false discovery rate (FDR) value (adjusted for the total number of protein comparisons) of *p*_(FDR)_ ≤ 0.05 (Benjamini and Hochberg, 1995; Storey and Tibshirani, 2003). Statistical comparisons are summarized in Table 6.

### Experiment 2: effect of age on expression of mGluR mRNA in mPFC subregions

#### Tissue micro-punching and RNA isolation

Animals were killed by rapid decapitation and whole brains were quickly extracted, frozen on dry ice, and stored at -80°C. Brains were equilibrated to -10°C in a cryostat and 360-µm sections were cut through the rostral-caudal extent of the frontal cortex. A 1-mm tissue biopsy punch tool was used to obtain samples from PrL and IL subregions of mPFC. Tissue punches were immediately transferred to homogenization buffer and total RNA was isolated using the RNEasy Plus Micro kit according to the manufacturer’s protocol (PN: 74034, QIAGEN). RNA concentration was determined with the use of a NanoDrop1000 (Thermo Scientific). The yield of RNA was consistent and reproduced across groups. The average RNA integrity number (RIN) determined by TapeStation (Agilent Biosciences) was 9.7, and no sample had a RIN lower than 9.

#### Reverse transcription and qPCR assay

From each sample, 100 ng of RNA was used to make cDNA using the RT^2^ PreAMP cDNA Synthesis kit (PN: 330451, QIAGEN). Then, cDNA targets were preamplified using the RT^2^ PreAMP PCR Mastermix and the RT^2^ PreAMP Pathway Primer Mix according to the manufacturer’s protocol (PN: PBR-152Z, QIAGEN). Relative gene expression was measured using RT^2^ Profiler low-density PCR plates preloaded with qPCR primers for genes encoding GABA- and glutamate-related targets (PN: PARN-152ZA, QIAGEN). This approach was taken to enable assessment of all mGluR subtypes in parallel. Thermal cycling and data collection was accomplished using an ABI Real-Time PCR 7300. Only RT-qPCR plates that passed the PCR array reproducibility, reverse transcription efficiency, and genomic DNA contamination quality control parameters set by QIAGEN’s preamplification methods (RT^2^ Profiler PCR Array Data Analysis v3.5) as well as those reactions that produced the predicted peak by melting temperature (T_m_) curve analysis were included in the final analyses. Consequently, final group sizes for PrL and IL analyses were *n* = 6 young and *n* = 12 aged.

#### Statistical analysis of genes

Each gene included in the RT-qPCR plates was cross-referenced with the Allen Brain Institute’s online *in situ* hybridization atlas (http://mouse.brain-map.org/) and those not expressed in mPFC were used to set the lowest cycle threshold (C_t_) considered detectable. Genes were normalized to the housekeeping gene *RPLP1*. This gene did not differ by age in either PrL or IL (mean group difference = 0.192 C_t_). After normalization, C_t_ values were transformed to percentage expression of young (i.e., mean level of young = 100%). Independent-samples *t* tests were used to compare expression of mGluR transcripts between young and aged samples in PrL and IL separately using the Benjamini–Hochberg method to correct for multiple comparisons with a FDR value (adjusted for the total number of gene comparisons) of *p*_(FDR)_ ≤ 0.00866 (Benjamini and Hochberg, 1995; Storey and Tibshirani, 2003). Statistical comparisons are summarized in Table 6.

### Experiment 3: contributions of mGluRs in PrL cortex to working memory

#### Surgical procedures

Rats were anesthetized with isofluorane gas and secured in a stereotaxic frame. Following a midline incision over the skull, the skin was retracted and holes were drilled in the skull for guide cannulae and stainless-steel anchoring screws. Bilateral guide cannulae (22 gauge, Plastics One) targeting the PrL subregion of the mPFC (AP: +2.7 mm from bregma, ML: ±0.7 mm from bregma, DV: -3.8 mm from the skull surface) were implanted and secured to the skull with the screws and dental cement. Stainless-steel obdurators were placed into the cannulae to minimize the risk of infection. Immediately after surgery, rats received subcutaneous injections of buprenorphine (1 mg/kg/d) and meloxicam (2 mg/kg/d). Buprenorphine was also administered 24 h postoperation, and meloxicam 48–72 h postoperation. A topical ointment was applied as needed to facilitate wound healing. Before behavioral procedures, rats received at least two weeks postsurgical recovery, with sutures removed after 10–14 d.

#### Behavioral testing apparatus

Behavioral testing was conducted in 8 identical standard rat behavioral test chambers (Coulbourn Instruments) with steel front and back walls, transparent Plexiglas side walls, and a floor composed of steel rods (0.4 cm in diameter) spaced 1.1 cm apart. Each test chamber was housed in a sound-attenuating cubicle and was equipped with a recessed food pellet delivery trough located 2 cm above the floor in the center of the front wall. The trough was fitted with a photobeam to detect head entries and a 1.12-W lamp for illumination. Food rewards consisted of 45-mg grain-based food pellets (PJAI; Test Diet). Two retractable levers were positioned to the left and right of the food trough (11 cm above the floor). An additional 1.12-W house light was mounted near the top of the rear wall of the sound-attenuating cubicle. A computer interfaced with the behavioral test chambers and equipped with Graphic State 3.01 software (Coulbourn Instruments) was used to control experiments and collect data.

### Delayed response task

#### Habituation and shaping of operant procedures

After rats recovered from surgery, they were food-restricted to 85% of their free-feeding weights. Rats progressed through three stages of shaping before starting the working memory assessment. These shaping procedures were designed to train rats to reliably press each of the two response levers, with each new stage beginning on the day immediately following completion of a previous stage. On the day before shaping stage 1, each rat was given five 45-mg food pellets in its home cage to reduce neophobia to the food reward used in the task. Shaping stage 1 consisted of a 64-min session of magazine training, involving 38 deliveries of a single food pellet with an intertrial interval of 100 ± 40 s. Shaping stage 2 consisted of lever press training, in which a single lever (left or right, counterbalanced across age groups) was extended and a press resulted in delivery of a single food pellet. After reaching a criterion of 50 lever presses in 30 min, rats were then trained on the opposite lever using the same procedures. During shaping stage 3, a nose poke into the food trough caused either the left or right lever (counterbalanced across trials in this stage of testing) to extend, and a press resulted in a single food pellet delivery. Rats were trained in shaping stage 3 until achieving 80 lever presses in a 30-min session.

#### Delayed response task procedures

**Figure 1.**
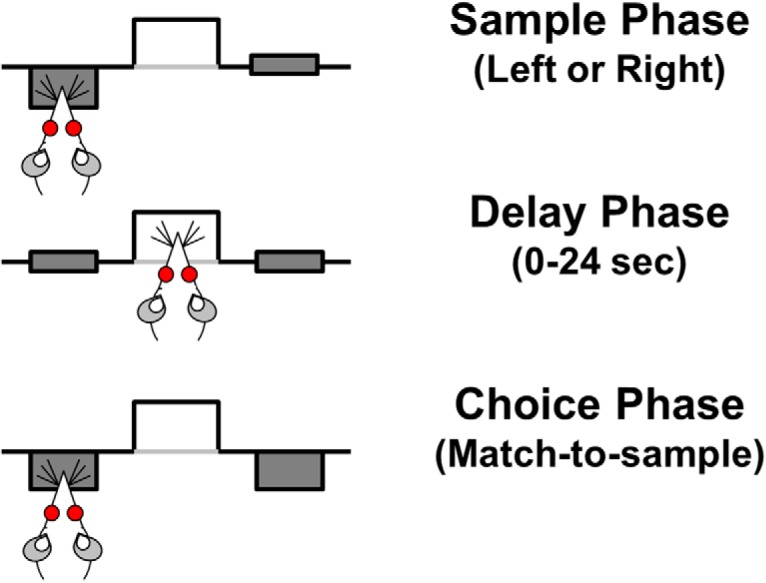
Schematic of delayed response working memory task. Each trial of the delayed response task includes three phases. During the sample phase, one lever (left or right, pseudo-randomly varied between pairs of trials) is extended into the chamber. The rat must press the extended lever to enter the variable duration “delay phase” (delays are pseudo-randomly varied from 0 to 24 s within each block of seven trials). During the delay, the rat must nose poke continuously into the centrally located food trough. The first nose poke emitted after the expiration of the predetermined delay timer initiates the choice phase wherein both levers (left and right) are extended into the chamber. The rat must remember and press the same lever that was extended during the sample phase to receive a food reward (a 45-mg food pellet), and this is scored as a correct choice. Pressing the other lever is scored as an incorrect choice and no food reward is delivered.

The task design was based on Sloan et al. (2006) and has been used by our lab previously to demonstrate working memory impairments in aged rats (Beas et al., 2013; Bañuelos et al., 2014; McQuail et al., 2016; Hernandez et al., 2017). Each 40-min session began with illumination of the house light, which remained illuminated throughout the entire session except during timeout periods. Rats received a single test session each day. Each trial in the task began with extension of a single “sample” lever into the chamber (Fig. 1). The sample lever (left or right) was randomly selected within each pair of trials to ensure equal representation of both levers across the test session. A press on the sample lever caused it to retract and initiated the delay interval. During the delay interval, rats were required to nose poke into the food trough to initiate the “choice phase.” Because there were no cues that signaled the duration of the delay period, and because delays were randomized across trials (making it impossible for rats to predict the end of the delay), this requirement resulted in rats nose poking continuously until the choice phase was initiated. This requirement that rats nose poke in the food trough during the delay interval also reduced the likelihood that they could employ non-mnemonic, “mediating” strategies (e.g., positioning themselves in front of the sample lever during the delay). The first nose poke executed after the delay interval expired initiated the choice phase by causing both levers to extend into the chamber. During the choice phase, a response on the same lever pressed during the sample phase was “correct” and resulted in retraction of both levers and delivery of a food pellet into the food trough, followed by a 5-s intertrial interval. A response on the opposite lever from that chosen during the sample phase was “incorrect” and resulted in retraction of both levers and initiation of a 5-s “timeout” period during which the house light was extinguished. Immediately following this timeout, the house light was re-illuminated and the next trial began (i.e., one lever was extended into the chamber for the “sample phase”).

During initial sessions in this task, there were no delays between the sample and choice phases, and a correction procedure was used such that the sample lever was repeated on the same side following an incorrect response, to reduce development of side biases. Once rats reached a criterion of 80% correct choices across a test session for two consecutive sessions, this correction procedure was discontinued and a set of seven delays was introduced. The presentation of delay durations was randomized within each block of seven trials, such that each delay was presented once within a block. On establishing >80% correct responses across two consecutive sessions in a “delay set,” rats were progressed to the next set, which contained increasingly longer delays (delay set 1: 0, 1, 2, 3, 4, 5, and 6 s; delay set 2: 0, 2, 4, 8, 12, and 16 s; delay set 3: 0, 2, 4, 8, 12, 18, and 24 s). Rats were trained on the last delay set until reaching stable baseline performance (defined as <10% variability across five consecutive days of training) at which point they were assigned to one of two drug groups used to test the effects of blockade of mGluR5 and mGluR2/3 (counterbalancing baseline performance across groups).

### Drug preparation and intracerebral micro-infusion

The selective noncompetitive mGluR5 antagonist (Anderson et al., 2002; Busse et al., 2004) 3-((2-methyl-4-thiazolyl)ethynyl)pyridine (MTEP; Tocris), was dissolved in artificial CSF (aCSF) at concentrations of 0.1, 0.3, and 1.0 µg per 0.5 µl. Doses were selected based on a previous study showing that intracerebral infusions targeting the mPFC with 15 nmol (3.5 µg) of MTEP per hemisphere prevented behavioral sensitization to cocaine (Timmer and Steketee, 2012). The mixed mGluR2/3 competitive antagonist (Kingston et al., 1998), (2S)-2-amino-2-[(1S,2S)-2-carboxycycloprop-1-yl]-3-(xanth-9-yl) propanoic acid (LY341495, Tocris), was dissolved in a 20% DMSO in aCSF solution at concentrations of 0.005, 0.05, and 0.5 µg per 0.5 µl. Doses were selected according to a previous study showing that intracerebral infusions targeting the amygdala with 0.3 µg of LY341495 per hemisphere blocked a Group II mGluR agonist-induced startle response (Walker et al., 2002).

After establishing baseline performance, rats were assigned to receive either MTEP or LY341495. Drug doses were administered using a randomized, within-subjects Latin square design such that each rat received each dose of drug and vehicle, with a 48-h washout period between successive infusions. Each infusion was administered by an experimenter who was blinded to the treatment conditions. Drugs were administered using 10-µl Hamilton syringes mounted on a Harvard Apparatus infusion pump (Pump 11 Elite, Harvard Apparatus) and connected via PE-20 tubing to micro-injectors (Plastics One), which extended 1 mm past the end of the guide cannulae. Each dose was delivered in a volume of 0.5 µl/hemisphere over a duration of 1 min, and injectors were left in place for one additional minute to allow for diffusion. Behavioral testing began 5 min postinfusion.

### Cannula placement histology

After completion of behavioral testing, rats were administered a lethal dose of Euthasol (sodium pentobarbital and phenytoin solution; Virbac) and perfused transcardially with a 4°C solution of 0.1 M PBS for 2 min, followed by 4% (w/v) paraformaldehyde in 0.1 M PBS for an additional 5 min. Brains were removed and postfixed for 24 h, then transferred to a 20% (w/v) sucrose solution in 0.1 M PBS for 3 d (all chemicals purchased from Fisher Scientific). Brains were sectioned at 40 µm using a cryostat maintained at -20°C, and slices were mounted on electrostatic glass slides. Brain sections were subsequently stained with thionin and coverslipped for verification of cannula placement under a compound light microscope. Injector tip coordinates were identified using a rat brain atlas (Paxinos and Watson, 2005). Off-target cannula placements required exclusion of *n* = 4 rats from the MTEP cohort (for finalized cannula placements, see Fig. 4*A*) and *n* = 1 rat from the LY341495 cohort (for finalized cannula placements, see Fig. 5*A*).

### Statistical analyses for behavioral pharmacology

Raw data files were exported from Graphic State software and compiled using a custom macro written for Microsoft Excel (Dr. Jonathan Lifshitz, University of Kentucky). Statistical analyses were conducted using SPSS 24.0 (IBM). Choice accuracy (the percentage of correct choices at each delay duration) was the primary measure of interest (Beas et al., 2013; Bañuelos et al., 2014; McQuail et al., 2016; Hernandez et al., 2017). Several additional measures were also compared to assess possible non-mnemonic effects on task performance (number of trials completed/session, see Figs. 4, 5; and latency to lever press during both the sample and choice phases of the trials, see Tables 4, 5). Choice accuracy was analyzed using a two-factor, repeated-measures ANOVA, with drug dose (four levels) and delay (seven levels) as within-subjects factors. The Huynh–Feldt correction was applied to correct for violations of sphericity. Significant main effects of dose or interactions between dose and delay were explored with a *post hoc*, two-factor, repeated-measures ANOVA to compare the effect of individual doses versus vehicle (two levels), with delay (seven levels) as an additional within-subjects factor in these analyses. To determine the effect of dose at specific delays, a *post hoc*, repeated-measures ANOVA was done with dose (four levels) as the within-subjects factor for each individual delay. Any significant effects of dose were followed up with a pairwise comparison using paired-samples *t* tests with Dunnett’s correction for multiple comparisons. The number of trials completed and lever press latencies were analyzed using a one-factor, repeated-measures ANOVA, with drug dose (four levels) as the within-subjects factor. The Huynh–Feldt correction was applied to correct for violations of sphericity. To determine whether there were carry-over or cumulative effects of successive PrL micro-infusions, choice accuracy on intervening wash-out days was analyzed by a repeated-measures ANOVA, using either dose delivered on the previous day or cumulative number of micro-infusions (four levels for each) as within-subjects factors. Statistical comparisons are summarized in Table 6.

## Results

### Experiment 1: expression of select mGluR proteins is reduced in aged PFC

**Figure 2.**
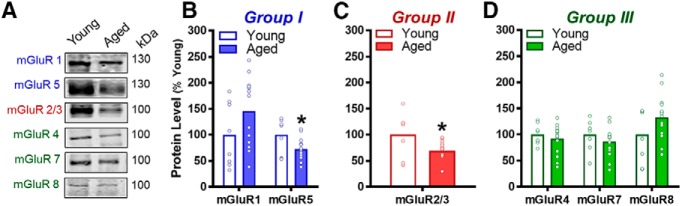
mGluR protein levels in mPFC of young and aged rats. ***A***, Representative images of immuno-reactive bands detected using mGluR subtype-selective antibodies in whole mPFC membrane homogenates prepared from young and aged rats. ***B***, Group I mGluRs (in blue). The protein level of mGluR5, but not mGluR1, was significantly lower in the mPFC of aged rats compared to young adults (*p < 0.05 vs young). ***C***, Group II mGluRs (in red). The protein level of mGluR2/3 was significantly lower in the mPFC of aged rats compared to young adults (*p < 0.05 vs young). ***D***, Group III mGluRs (in green). There were no significant changes to the protein levels of Group III mGluRs of aged rats compared to young adults (*p* > 0.05 vs young). ***B–D***, Mean protein level (transformed to “% of young” after normalizing integrated intensity to α-tubulin, *y*-axis; see Table 2 for normalized, untransformed data) is plotted as a function of mGluR subtype (*x*-axis) and age group (separate bars; *n* = 7-8 young and *n* = 15 aged). Open circles represent values for individual rats and bars represent group means.

Group I mGluRs are largely localized to postsynaptic sites and include mGluR1 and mGluR5. Expression of mGluR1 in the mPFC did not reliably differ between young adult and aged rats (*t*_(21)_ = -1.670, *p* = 0.110; Fig. 2*B*); however, expression of mGluR5 was significantly decreased in aged relative to young (*t*_(20)_ = 2.407, *p* = 0.026; Fig. 2*B*). Group II mGluRs are comprised of mGluR2 and mGluR3, and these receptors have been identified on both pre- and postsynaptic sites (Tanabe et al., 1993; Okamoto et al., 1994; Mannaioni et al., 2001; Sansig et al., 2001; Tyszkiewicz et al., 2004; Hagenston et al., 2008; Niswender and Conn, 2010; Arnsten et al., 2012; Jin et al., 2017). Although antibodies do not distinguish between these receptors, expression of mGluR2/3 was also significantly lower in the aged mPFC compared to young (*t*_(20)_ = 2.366, *p* = 0.028; Fig. 2*C*). In contrast, expression of the largely presynaptic Group III receptors, mGluR4, mGluR7, and mGluR8, was unchanged with age (*t*s_(21)_ = -1.650–1.134, *p*s = 0.114–0.459; Fig. 2*D*). See Table 2 for normalized data (not expressed as percentage of young) and note that mGluR6 is not expressed in brain and thus was not analyzed.

**Table 2.** Age effects on protein levels in the mPFC (untransformed normalized protein values)

Protein	Young	Aged	Δ from young	*t*_(20–21)_, *p* value
mGluR1	20,307.25 ± 4395.02	29,617.20 ± 3327.26	9309.95	*t*_(21)_ *=* -1.67, *p* = 0.110
mGluR5	82,644.20 ± 10,450.19	59,508.56 ± 4488.32	-23,135.65	*t*_(20)_ = 2.407, *p* = 0.026
mGluR2/3	725,810.54 ± 119,325.75	498,933.86 ± 36,495.67	-226,876.68	*t*_(20)_ = 2.366, *p* = 0.028
mGluR4	50,511.34 ± 3501.51	46,345.35 ± 3556.84	-4165.99	*t*_(21)_ = 0.754, *p* = 0.459
mGluR7	1,389,307.48 ± 141,709.53	1,199,490.29 ± 96,296.40	-18,9817.20	*t*_(21)_ = 1.134, *p* = 0.270
mGluR8	5268.93 ± 941.36	6971.13 ± 565.52	1702.20	*t*_(21)_ = -1.649, *p* = 0.114

### Experiment 2: age-associated reductions in mGluR mRNA expression are PFC subregion specific

**Figure 3.**
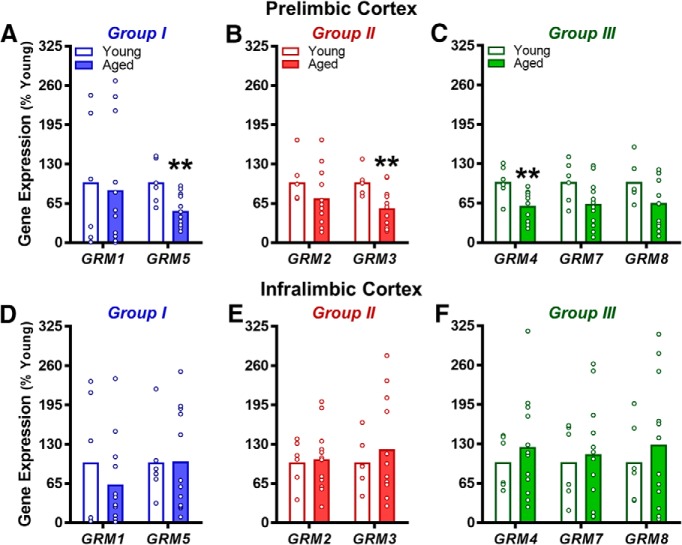
mGluR gene transcript expression in the PrL and IL of young and aged rats. ***A***, Group I mGluRs (in blue). Expression of *GRM5*, but not *GRM1*, was significantly lower in the PrL subregion of aged rats compared to young adults (**p < 0.01 vs young). ***B***, Group II mGluRs (in red). Expression of *GRM3*, but not *GRM2*, was significantly lower in the PrL of aged rats compared to young adults (**p < 0.01 vs young). ***C***, Group III mGluRs (in green). Expression of *GRM4*, but not *GRM7* or *GRM8*, was significantly lower in the PrL of aged rats compared to young adults (**p < 0.01 vs young). ***D–F***, Gene expression was not significantly different between young adult and aged rats in the IL subregion. In all panels, mean gene expression (transformed to “% of young” after normalizing raw C_t_ values to *RPLP1*; *y*-axis) is plotted as a function of gene (*x*-axis) and age group (separate bars; *n* = 6 young and *n* = 12 aged). Open circles represent values for individual rats and bars represent group means.

To confirm and extend the significant findings observed at the level of mGluR protein, complementary analyses were performed to measure expression of mRNAs that encode for these receptors in a second cohort of young and aged rats. Relative to Western blotting, PCR requires comparatively smaller sample quantities, allowing for differentiation of the mPFC into PrL and IL subregions for discrete analyses. Further, unlike commercial antibodies, PCR primer probes can distinguish between *GRM2* and *GRM3*. Table 3 and Figure 3 summarize statistical comparisons of mGluR gene expression in young and aged rats using this analysis. In agreement with the data from Western blots, both *GRM5* and *GRM3* were significantly reduced in aged PrL. Expression of *GRM4* also was reliably reduced in aged PrL compared to young. Expression of *GRM7*, *GRM8*, *GRM1*, and *GRM2* was preserved in aged PrL relative to young. In contrast to selective mGluR mRNA reductions in PrL, expression of mRNA for all mGluR subtypes did not differ as a function of age in IL. See Table 3 for normalized data (not expressed as percentage of young).

**Table 3.** Age effects on gene expression in the PrL and the IL (untransformed normalized RNA values)

Gene	Subregion	Young	Aged	Δ from young	*t*_(16)_, *p* value
GRM1	PrL IL	0.002235 ± 0.000978 0.002033 ± 0.000908	0.001937 ± 0.000645 0.001286 ± 0.000427	-0.000299 -0.000747	*t*_(16)_ = 0.261, *p* = 0.798 *t*_(16)_ = -0.854, *p* = 0.406
GRM5	PrL IL	0.054026 ± 0.007805 0.041180 ± 0.010800	0.028365 ± 0.004163 0.041917 ± 0.009986	-0.025661 0.000737	*t*_(16)_ = 3.200, *p* = 0.006 *t*_(16)_ = -0.046, *p* = 0.964
GRM2	PrL IL	0.028930 ± 0.004422 0.013810 ± 0.002117	0.021187 ± 0.003913 0.014570 ± 0.002094	-0.007744 0.000760	*t*_(16)_ = 1.213, *p* = 0.243 *t*_(16)_ = -0.230, *p* = 0.823
GRM3	PrL IL	0.056213 ± 0.004957 0.041953 ± 0.007275	0.032051 ± 0.005106 0.050947 ± 0.010043	-0.024162 0.008994	*t*_(16)_ = 2.990, *p* = 0.009 *t*_(16)_ = -0.589, *p* = 0.479
GRM4	PrL IL	0.009190 ± 0.001015 0.006980 ± 0.001232	0.005563 ± 0.000669 0.008730 ± 0.001687	-0.003627 0.001750	*t*_(16)_ = 3.058, *p* = 0.008 *t*_(16)_ = -0.682, *p* = 0.505
GRM7	PrL IL	0.040217 ± 0.005571 0.029601 ± 0.007437	0.025655 ± 0.004430 0.033461 ± 0.007364	-0.014562 0.003861	*t*_(16)_ = 1.963, *p* = 0.067 *t*_(16)_ = -0.329, *p* = 0.746
GRM8	PrL IL	0.016475 ± 0.002306 0.010927 ± 0.002872	0.010738 ± 0.001938 0.014094 ± 0.003378	-0.005737 0.003167	*t*_(16)_ = 1.793, *p* = 0.092 *t*_(16)_ = -0.605, *p* = 0.554

### Experiment 3: mGluR3 and mGluR5 in PrL are necessary for normal working memory

Data from mGluR protein and gene expression studies converge to potentially implicate reductions in specific mGluRs in the age-associated decline of cognitive processes supported by the mPFC. Specifically, findings from experiments 1 and 2, together with a large literature implicating PFC in working memory, suggest that the decline of mGluR5 and mGluR3 in the aged PrL might contribute to the well-documented impairments in this aspect of cognition that emerge in later life (Oscar-Berman and Bonner, 1985; Dunnett et al., 1988; Rapp and Amaral, 1989; Bachevalier et al., 1991; Lamar and Resnick, 2004; Beas et al., 2013; McQuail et al., 2016; Hernandez et al., 2017). The final experiments in this study were designed to determine whether these mGluR reductions could be sufficient to impact working memory performance. In these studies, two cohorts of young adult rats were used to test the effects of intra-PrL micro-infusions of selective mGluR antagonists targeting mGluR5 or mGluR2/3 on performance in a delayed response task that evaluates working memory.

**Figure 4.**
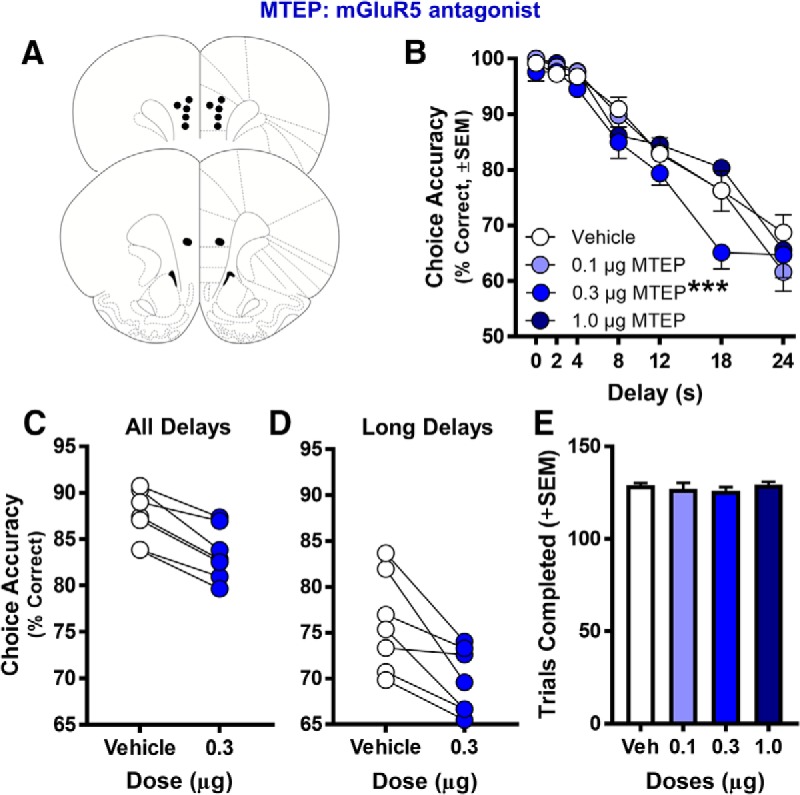
Effect of micro-infusing MTEP (mGluR5 antagonist) into PrL cortex on performance in the delayed response working memory task. ***A***, Histologically verified placements of injector tips used to micro-infuse the mGluR5 antagonist MTEP into the PrL cortex of young adult rats before testing in the delayed response task (*n* = 7 young rats). ***B***, Micro-infusion of 0.3-µg MTEP significantly reduced choice accuracy relative to vehicle (*n* = 7; ***p < 0.05 vs vehicle, main effect of dose). ***C***, *Post hoc* analysis comparing 0.3-µg dose of MTEP to vehicle. The 0.3-µg dose of MTEP impaired performance across all delays in all rats compared to vehicle performance, *p* < 0.001. ***D***, The 0.3-µg dose of MTEP impaired performance at long delays (12–24 s) in all rats compared to vehicle performance. ***E***, The number of trials completed did not change as a function of MTEP dose. In ***A***, placements are mapped to standardized coronal sections corresponding to +2.70 and +3.20 mm from bregma according to the atlas of Paxinos and Watson (2005). In ***B***, mean choice accuracy (*y*-axis) is plotted as a function of delay (*x*-axis) and dose (symbols/lines; refer to legend for specific dose). In ***C***, ***D***, mean choice accuracy (collapsed across all delays in ***C*** and long delays (12–24 s) in ***D***; *y*-axis) is plotted as a function of the 0.3-µg dose of MTEP (*x*-axis; symbols/lines). Error bars represent the SEM.

In the first cohort of rats, the effects on working memory resulting from blockade of the group I receptor mGluR5 were tested using the selective mGluR5 antagonist MTEP. Intra-PrL infusion of MTEP significantly impaired choice accuracy (Fig. 4*B*; main effect of dose: *F*_(3,18)_ = 3.176, *p* = 0.049; dose × delay interaction: *F*_(18,108)_ = 1.096, *p* = 0.366). *Post hoc* comparisons probing individual doses relative to vehicle indicated that the 0.3-µg dose of MTEP reliably impaired choice accuracy (Fig. 4*C*,*D*; main effect of dose: *F*_(1,6)_ = 54.178, *p* = 0.0001; dose × delay: *F*_(6,36)_ = 1.388, *p* = 0.246), whereas performance under other doses did not significantly differ from vehicle (main effects of dose *F*s_(1,6)_ = 0.024–2.44, *p*s = 0.639–0.882; dose × delay interactions: *F*s_(6,36)_ = 0.765–1.186, *p*s = 0.336–0.602). To evaluate potential carry-over effects of the drug micro-infusions, performance on intervening days of the drug schedule (i.e., wash-out days) was also evaluated. Performance on these days did not differ as a function of either the dose administered on the previous day (main effect of prior day’s dose: *F*_(3,18)_ = 0.239, *p* = 0.868; data not shown) or as a function of infusion day (main effect of infusion day: *F*_(3,18)_ = 1.205, *p* = 0.334; data not shown), indicating there were no residual effects of MTEP on task performance that carried forward to subsequent drug days, nor deleterious effects on task performance from the cumulative effects of successive micro-infusions. To determine whether MTEP influenced non-mnemonic aspects of task performance, several additional measures were also assessed. Analysis of the total number of trials completed per session revealed no effect of MTEP dose (Fig. 4*E*; *F*_(3,18)_ = 0.571, *p* = 0.642). Additional analyses of lever press response latencies revealed no effects of MTEP dose during either the sample (*F*_(3,18)_ = 1.149, *p* = 0.356) or choice (matching) (*F*_(3,18)_ = 2.55, *p* = 0.088) phases of the task (Table 4). These data suggest that the effects of MTEP on delayed response choice accuracy were not secondary to effects on motivation or general task performance.

**Table 4.** Effects of MTEP (mGluR5 antagonist) on response latencies

Response latency	Dose	Mean (ms)	SE	*N*
Sample phase *F*_(3,18)_ = 1.149, *p* = 0.356	Vehicle	1729.74	103.44	7
	0.1 µg	2213.25	388.26	7
	0.3 µg	2422.28	305.92	7
	1.0 µg	1893.21	224.53	7
Matching phase *F*_(3,18)_ = 2.55, *p* = 0.088	Vehicle	1013.31	82.56	7
	0.1 µg	1014.81	90.96	7
	0.3 µg	1017.94	66.11	7
	1.0 µg	942.91	91.39	7

**Figure 5.**
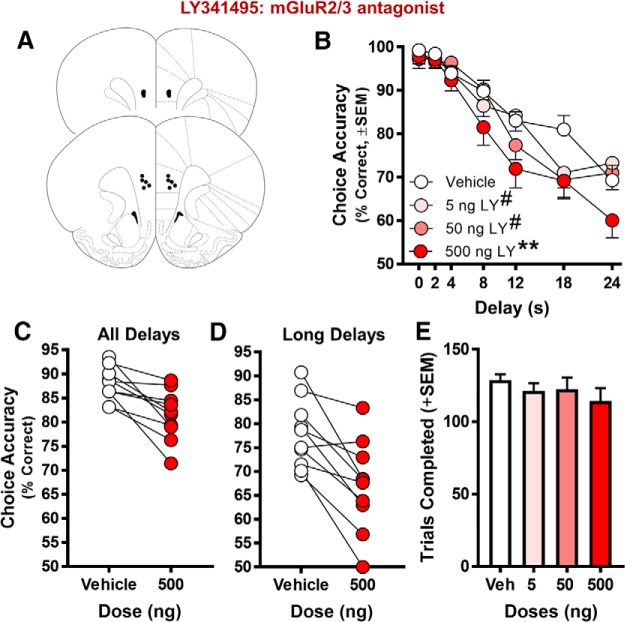
Effect of micro-infusing LY341495 (mGluR2/3 antagonist) into PrL cortex on performance in the delayed response working memory task. ***A***, Histologically verified placements of injector tips used to micro-infuse the mGluR2/3 antagonist LY341495 into the PrL cortex of young adult rats before testing in the delayed response task (*n* = 10 young rats). ***B***, Microinfusion of LY341495 significantly reduced choice accuracy relative to vehicle at all doses tested (*n* = 10; **p < 0.05 vs vehicle, main effect of dose; #*p* < 0.05 vs vehicle, dose × delay interaction). ***C***, *Post hoc* analysis comparing 500-ng dose of LY341495 to vehicle. The 500-ng dose impaired performance across all delays in all rats compared to vehicle performance, *p* < 0.01. ***D***, Micro-infusion of 500-ng LY341495 impaired performance at long delays (12–24 s) compared to vehicle performance. ***E***, The number of trials completed did not change as a function of dose. In ***A***, placements are mapped to standardized coronal sections corresponding to +2.70 and +3.20 mm from bregma according to the atlas of Paxinos and Watson (2005). In ***B***, mean choice accuracy (*y*-axis) is plotted as a function of delay (*x*-axis) and dose (symbols/lines; refer to legend for specific dose). In ***C***, ***D***, mean choice accuracy (collapsed across all delays in ***C*** and long delays (12–24 s) in ***D***; *y*-axis) is plotted as a function of the 500-ng dose of LY341495 (*x*-axis; symbols/lines). Error bars represent the SEM.

In the second cohort of rats used for behavioral pharmacology, the effects on working memory resulting from blockade of group II receptors (mGluR2/3) were tested using LY341495. A main effect of dose (*F*_(3,27)_ = 4.778, *p* = 0.008) and a significant dose × delay interaction (*F*_(18,162)_ = 2.083, *p* = 0.009) on choice accuracy were observed following LY341495 administration (Fig. 5*B*). *Post hoc* analyses comparing individual doses to vehicle determined that all doses significantly impaired performance (5 ng, main effect of dose: *F*_(1,9)_ = 0.570, *p* = 0.470; dose × delay interaction: *F*_(6,54)_ = 2.834, *p* = 0.018; 50 ng, main effect of dose: *F*_(1,9)_ = 1.895, *p* = 0.202; dose × delay interaction: *F*_(6,54)_ = 2.887, *p* = 0.029; 500 ng, main effect of dose: *F*_(1,9)_ = 14.911, *p* = 0.004, dose × delay: *F*_(6,54)_ = 1.793, *p* = 0.118, Fig. 5*C*,*D*). A further *post hoc* analysis on the effect of dose at each delay revealed significant main effects of dose at both the 18-s delay (*F*_(3,27)_ = 5.009, *p* = 0.007; veh > 5 ng: *t*_(9)_ = 2.501, *p* = 0.034; veh > 50 ng: *t*_(9)_ = 3.212, *p* = 0.011; veh > 500 ng: *t*_(9)_ = 2.596, *p* = 0.029) and 24-s delay (*F*_(3,27)_ = 3.570, *p* = 0.027; veh > 500 ng: *t*_(9)_ = 2.402, *p* = 0.040; see Table 6 for summary of all *post hoc* analyses). As with MTEP, there was no residual effect of LY341495 on task performance on the wash-out days following drug infusion (main effect of prior day’s dose: *F*_(3,27)_ = 1.341, *p* = 0.282; data not shown) nor did the cumulative number of micro-infusions influence performance (main effect of infusion day: *F*_(3,27)_ = 0.276, *p* = 0.842; data not shown). Finally, LY341495 had no effects on either the number of trials completed (*F*_(3,27)_ = 2.422, *p* = 0.088; Fig. 5*E*) or lever press response latencies (sample phase: *F*_(3,27)_ = 2.017, *p* = 0.185; choice (matching) phase *F*_(3,27)_ = 1.000, *p* = 0.408; Table 5).

**Table 5.** Effects of LY341495 (mGluR2/3 antagonist) on response latencies

Response latency	Dose	Mean (ms)	SE	*N*
Sample phase *F*_(3,27)_ = 2.017, *p* = 0.185	Vehicle	1915.99	447.33	10
	5 ng	3014.86	971.06	10
	50 ng	2948.58	1379.83	10
	500 ng	4907.86	2351.06	10
Matching phase *F*_(3,27)_ = 1.0, *p* = 0.408	Vehicle	1059.35	106.66	10
	5 ng	1191.25	120.88	10
	50 ng	1108.37	83.11	10
	500 ng	1173.37	104.58	10

**Table 6.** Summary of statistical analyses

	Measure	Factor(s)	Level(s)	Data structure: normality tests Kolmogorov–Smirnov (*t* test); Mauchly’s sphericity (ANOVAs)	Type of test	Statistical value	*p* value	Effect size: Cohen's *d* (*t* test); partial η^2^ (ANOVA)	Observed power
a	mGluR1 protein level in whole mPFC	Age	2	Normal	*t* test (FDR-corrected)	*t* = -1.670	0.110	0.740	0.380
b	mGluR5 protein level in whole mPFC	Age	2	Normal	*t* test (FDR-corrected)	*t* = 2.407	0.026	1.000	0.449
c	mGluR2/3 protein level in whole mPFC	Age	2	Normal	*t* test (FDR-corrected)	*t* = 2.366	0.028	0.928	0.350
d	mGluR4 protein level in whole mPFC	Age	2	Normal	*t* test (FDR-corrected)	*t* = 0.754	0.459	0.347	0.064
e	mGluR7 protein level in whole mPFC	Age	2	Normal	*t* test (FDR-corrected)	*t* = 1.134	0.270	0.490	0.192
f	mGluR8 protein level in whole mPFC	Age	2	Normal	*t* test (FDR-corrected)	*t* = -1.650	0.114	0.698	0.310
g	GRM1 gene expression in PrL	Age	2	Normal	*t* test (FDR-corrected)	*t* = 0.261	0.798	0.129	0.058
h	GRM5 gene expression in PrL	Age	2	Normal	*t* test (FDR-corrected)	*t* = 3.200	0.006	1.515	0.767
i	GRM2 gene expression in PrL	Age	2	Normal	*t* test (FDR-corrected)	*t* = 1.213	0.243	0.631	0.209
j	GRM3 gene expression in PrL	Age	2	Normal	*t* test (FDR-corrected)	*t* = 2.990	0.009	1.593	0.781
k	GRM4 gene expression in PrL	Age	2	Normal	*t* test (FDR-corrected)	*t* = 3.058	0.008	1.509	0.831
l	GRM7 gene expression in PrL	Age	2	Normal	*t* test (FDR-corrected)	*t* = 1.963	0.067	1.002	0.476
m	GRM8 gene expression in PrL	Age	2	Normal	*t* test (FDR-corrected)	*t* = 1.793	0.092	0.923	0.402
n	GRM1 gene expression in IL	Age	2	Non-normal	*t* test (FDR-corrected)	*t* = -0.854	0.406	0.395	0.103
o	GRM5 gene expression in IL	Age	2	Normal	*t* test (FDR-corrected)	*t* = -0.046	0.964	0.024	0.051
p	GRM2 gene expression in IL	Age	2	Normal	*t* test (FDR-corrected)	*t* = -0.228	0.823	0.121	0.055
q	GRM3 gene expression in IL	Age	2	Normal	*t* test (FDR-corrected)	*t* = -0.589	0.479	0.325	0.082
r	GRM4 gene expression in IL	Age	2	Normal	*t* test (FDR-corrected)	*t* = -0.682	0.505	0.376	0.093
s	GRM7 gene expression in IL	Age	2	Normal	*t* test (FDR-corrected)	*t* = -0.329	0.746	0.174	0.061
t	GRM8 gene expression in IL	Age	2	Normal	*t* test (FDR-corrected)	*t* = -0.605	0.554	0.328	0.085
u	MTEP choice accuracy (all doses)	Dose	4	Sphericity assumed	Repeated-measures ANOVA	*F* = 3.176	0.049	0.364	0.634
		Dose by delay	4*7	Sphericity assumed	Repeated-measures ANOVA	*F* = 1.096	0.366	0.154	0.720
		Delay	7	Sphericity assumed	Repeated- measures ANOVA	*F* = 87.404	0.000	0.936	1.000
v	MTEP choice accuracy (0.1-µg dose)	Dose	2	Sphericity assumed	Repeated- measures ANOVA	*F* = 2.44	0.639	0.039	0.070
		Dose by delay	2*7	Sphericity violated: Huynh–Feldt corrected	Repeated- measures ANOVA	*F* = 0.765	0.602	0.113	0.262
		Delay	7	Sphericity assumed	Repeated- measures ANOVA	*F* = 56.882	0.000	0.905	1.000
w	MTEP choice accuracy (0.3-µg dose)	Dose	2	Sphericity assumed	Repeated- measures ANOVA	*F* = 54.178	0.000	0.900	1.000
		Dose by delay	2*7	Sphericity assumed	Repeated- measures ANOVA	*F* = 1.388	0.246	0.188	0.471
		Delay	7	Sphericity assumed	Repeated- measures ANOVA	*F* = 55.700	0.000	0.903	1.000
x	MTEP choice accuracy (1.0-µg dose)	Dose	2	Sphericity assumed	Repeated- measures ANOVA	*F* = 0.024	0.882	0.004	0.052
		Dose by delay	2*7	Sphericity assumed	Repeated- measures ANOVA	*F* = 1.186	0.336	0.165	0.404
		Delay	7	Sphericity violated: Huynh–Feldt corrected	Repeated- measures ANOVA	*F* = 28.045	0.000	0.824	1.000
y	MTEP Trials	Dose	4	Sphericity assumed	Repeated- measures ANOVA	*F* = 0.571	0.642	0.087	0.145
z	MTEP response latency (matching phase)	Dose	4	Sphericity assumed	Repeated- measures ANOVA	*F* = 2.550	0.088	0.298	0.531
aa	MTEP response latency (sample phase)	Dose	4	Sphericity assumed	Repeated- measures ANOVA	*F* = 1.149	0.356	0.161	0.257
bb	MTEP carry-over effects (washout days, all doses)	Day	4	Sphericity assumed	Repeated- measures ANOVA	*F* = 0.239	0.868	0.038	0.087
cc	MTEP injections (all doses)	Injection	4	Sphericity violated: Huynh–Feldt corrected	Repeated- measures ANOVA	*F* = 1.205	0.334	0.167	0.269
dd	LY341495 choice accuracy (all doses)	Dose	4	Sphericity assumed	Repeated- measures ANOVA	*F* = 4.778	0.008	0.347	0.853
		Dose by delay	4*7	Sphericity assumed	Repeated- measures ANOVA	*F* = 2.083	0.009	0.188	0.978
		Delay	7	Sphericity violated: Huynh–Feldt corrected	Repeated- measures ANOVA	*F* = 49.091	0.000	0.845	1.000
ee	LY341495 choice accuracy (5-ng dose)	Dose	2	Sphericity assumed	Repeated- measures ANOVA	*F* = 0.570	0.470	0.060	0.104
		Dose by delay	2*7	Sphericity assumed	Repeated- measures ANOVA	*F* = 2.834	0.018	0.239	0.847
		Delay	7	Sphericity violated: Huynh–Feldt corrected	Repeated- measures ANOVA	*F* = 29.521	0.000	0.766	1.000
ff	LY341495 choice accuracy (50-ng dose)	Dose	2	Sphericity assumed	Repeated- measures ANOVA	*F* = 1.895	0.202	0.174	0.234
		Dose by delay	2*7	Sphericity violated: Huynh–Feldt corrected	Repeated- measures ANOVA	*F* = 2.887	0.029	0.243	0.855
		Delay	7	Sphericity violated: Huynh–Feldt corrected	Repeated- measures ANOVA	*F* = 41.188	0.000	0.821	1.000
gg	LY341495 choice accuracy (500-ng dose)	Dose	2	Sphericity assumed	Repeated- measures ANOVA	*F* = 14.911	0.004	0.624	0.929
		Dose by delay	2*7	Sphericity assumed	Repeated- measures ANOVA	*F* = 1.793	0.118	0.166	0.623
		Delay	7	Sphericity violated: Huynh–Feldt corrected	Repeated- measures ANOVA	*F* = 37.902	0.000	0.808	1.000
hh	LY341495 choice accuracy (0-s delay)	Dose	4	Sphericity violated: Huynh–Feldt corrected	Repeated- measures ANOVA	*F* = 0.617	0.512	0.064	0.124
ii	LY341495 choice accuracy (2-s delay)	Dose	4	Sphericity assumed	Repeated- measures ANOVA	*F* = 0.527	0.668	0.055	0.143
jj	LY341495 choice accuracy (4-s delay)	Dose	4	Sphericity assumed	Repeated- measures ANOVA	*F* = 0.267	0.848	0.029	0.094
kk	LY341495 choice accuracy (8-s delay)	Dose	4	Sphericity assumed	Repeated- measures ANOVA	*F* = 2.239	0.107	0.199	0.504
ll	LY341495 choice accuracy (12-s delay)	Dose	4	Sphericity assumed	Repeated- measures ANOVA	*F* = 2.911	0.053	0.244	0.627
mm	LY341495 choice accuracy (18-s delay)	Dose (all doses)	4	Sphericity assumed	Repeated- measures ANOVA	*F* = 5.009	0.007	0.358	0.870
		Dose (Veh vs 5 ng)	2	Normal	*Post hoc* paired-samples *t* test (Dunnett-corrected)	*t* = 2.501	0.034	0.945	0.260
		Dose (Veh vs 50 ng)	2	Normal	*Post hoc* paired-samples *t* test (Dunnett-corrected)	*t* = 3.212	0.011	0.956	0.265
		Dose (Veh vs 500 ng)	2	Normal	*Post hoc* paired-samples *t* test (Dunnett-corrected)	*t* = 2.596	0.029	1.069	0.318
nn	LY341495 choice accuracy (24-s delay)	Dose (all doses)	4	Sphericity assumed	Repeated- measures ANOVA	*F* = 3.57	0.027	0.284	0.725
		Dose (Veh vs 5 ng)	2	Normal	*Post hoc* paired-samples *t* test (Dunnett-corrected)	*t* = -1.041	0.325	0.364	0.073
		Dose (Veh vs 50 ng)	2	Normal	*Post hoc* paired-samples *t* test (Dunnett-corrected)	*t* = -0.347	0.736	0.146	0.039
		Dose (Veh vs 500 ng)	2	Normal	*Post hoc* paired-samples *t* test (Dunnett-corrected)	*t* = 2.402	0.040	0.778	0.190
oo	LY341495 trials	Dose	4	Sphericity assumed	Repeated- measures ANOVA	*F* = 2.422	0.088	0.212	0.540
pp	LY341495 response latency (matching phase)	Dose	4	Sphericity assumed	Repeated- measures ANOVA	*F* = 1.000	0.408	0.100	0.242
qq	LY341495 response latency (sample phase)	Dose	4	Sphericity violated: Huynh–Feldt corrected	Repeated- measures ANOVA	*F* = 2.017	0.185	0.183	0.460
rr	LY341495 Carry-over effects (washout days, all doses)	Day	4	Sphericity assumed	Repeated- measures ANOVA	*F* = 1.341	0.282	0.130	0.316
ss	LY341495 injections (all doses)	Injection	4	Sphericity assumed	Repeated- measures ANOVA	*F* = 0.276	0.842	0.030	0.096

## Discussion

The goal of this study was to compare expression of all known mGluR subtypes in the mPFC between fully mature young adult and aged rats, and to differentiate the effects of selective mGluR antagonists in mPFC on normal working memory. Experiments 1 and 2 were directed at evaluating both protein and mRNA expression of mGluRs in aging, using complementary methodology in independent cohorts of young adult and aged rats. The biochemical analysis indicated that expression of mGluR2/3 and mGluR5 was reliably reduced with age. While arguably the protein expression data provide the most functionally-relevant information regarding the influence of age on these receptors, current antibodies do not distinguish between several of the mGluRs. Moreover, the quantity of tissue required for reliable protein assessment makes it difficult to restrict the analysis to anatomically and functionally distinct mPFC subregions (specifically PrL and IL). To provide confirmatory and complementary data regarding subregional mGluR expression, the mRNA transcripts for each receptor were probed using low density, PCR-based arrays that included genes for all known mGluRs (*GRM1-8*). This strategy allowed expression of *GRM2* and *GRM3* to be differentiated in addition to enabling the effects of age to be isolated in PrL and IL subregions. This approach corroborated the loss of mGluR5 and specified that loss of mGluR2/3 detected at the protein level is likely attributable to lower *GRM3* expression. Importantly, these data agree with postmortem studies that were prospectively designed to compare mGluR expression in PFC between schizophrenia patients and healthy controls, but incidentally observed that expression of both mGluR2/3 and mGluR5 are negatively correlated with age (Crook et al., 2002; Corti et al., 2011; Frank et al., 2011).

At the mRNA level, the loss of mGluRs was localized to the PrL subregion of the mPFC. The significance of this subregion-specific effect may pertain to unique characteristics of the PrL that support mnemonic function, due in part to extensive interconnections with other cortico-limbic brain regions (Seamans et al., 1995; Vertes, 2004, 2006; Cassaday et al., 2014). In contrast, the neighboring IL subregion, which exhibited no significant age-related changes in expression of mGluR genes, is known to connect more extensively with subcortical targets to regulate autonomic viscero-motor processes (Vertes, 2004, 2006). Indeed, pharmacological inactivation, or optogenetic manipulation localized to the PrL demonstrates that this mPFC subregion is required for behaviors that engage working memory (Sierra-Mercado et al., 2011; Gilmartin et al., 2013; Kim et al., 2016; Levin et al., 2017).

In addition to *GRM3* and *GRM5*, the analysis of mRNA expression also revealed a reliable age-related reduction in *GRM4* in PrL, although mPFC mGluR4 protein expression did not differ as a function of age. While implicated in psychiatric disorders (Woźniak et al., 2016; Isherwood et al., 2017), learning and memory (Davis et al., 2013; Iscru et al., 2013), and neurodegenerative disease (Niswender et al., 2016), the role of GRM4 in cognition is not well understood. Ligands targeting mGluR4 are currently unavailable, but important future work includes the exploration of this receptor in relation to working memory and other PFC-mediated cognitive functions in aging and disease states.

### Selective blockade of mGluR5 and mGluR2/3 impairs working memory performance

The second major finding of this study is that mGluRs in the PrL contribute to optimal working memory function. The few previous studies using systemic administration of mGluR5- or mGluR2/3-directed ligands have produced varied conclusions regarding the contributions of these receptors to working memory (Aultman and Moghaddam, 2001; Campbell et al., 2004; Homayoun et al., 2004; Novitskaya et al., 2010). Although these receptors are highly expressed in the PFC, systemic drug administration cannot isolate the contribution of PFC mGluRs to working memory as they are also present in other brain regions that contribute to diverse aspects of cognition (Ferraguti and Shigemoto, 2006; Gravius et al., 2010). To determine whether signaling via mGluRs in the mPFC, and more specifically the PrL, is necessary for working memory, we investigated the effects of micro-infusing subtype-selective antagonists into the PrL during delayed response task performance.

In the current study, the highly selective mGluR5 antagonist MTEP impaired delayed response accuracy without influencing non-mnemonic aspects of performance (number of trials completed or response latencies). The fact that this impairment, as well as that induced by mGluR2/3 blockade, was delay-dependent is consistent with the interpretation that mGluR blockade in mPFC specifically impaired working memory. While rats can use mediating strategies that circumvent mnemonic demands on delayed response tasks (e.g., leaning their body toward the correct lever while nose poking in the food trough; Herremans et al., 1996; Chudasama and Muir, 1997), such strategies would not be expected to produce a robust pattern of declining accuracy with increasing delays. In fact, this pattern of declining accuracy with increasing delays is reliably observed, even under baseline conditions (Beas et al., 2013; Bañuelos et al., 2014; McQuail et al., 2016; Hernandez et al., 2017).

The importance of mGluR5 to working memory may relate to its ability to increase excitability and firing of mPFC pyramidal neurons in response to synaptic stimulation (Homayoun and Moghaddam, 2006; Lecourtier et al., 2007; Sidiropoulou et al., 2009). Specifically, sustained activity of neurons in the PFC during delays interposed between stimulus perception and response initiation is widely considered to be the physiologic basis for temporary storage of information in working memory (Goldman-Rakic, 1995). Ionotropic NMDARs are essential for persistent firing and working memory (Wang et al., 2013; McQuail et al., 2016) and mGluR5 may support these processes by potentiating NMDAR currents (Mannaioni et al., 2001). Consistent with the view that mGluR5 exerts its effects on working memory via interactions with NMDARs are data showing that mGluR5 blockade exacerbates working memory impairments induced by NMDAR antagonists, including phencyclidine (PCP) and MK-801 (Campbell et al., 2004; Homayoun et al., 2004). Diminished contributions from mGluR5, as in normal aging or following pharmacological blockade, may shift glutamate signaling toward preserved binding sites on mGluR1 that stimulate postsynaptic changes that are less advantageous to working memory. Specifically, mGluR1 stimulates release of Ca^2+^ from intracellular stores (Mannaioni et al., 2001), and such mGluR-mediated mobilization of intracellular Ca^2+^ is associated with mixed effects on PFC neuron excitability (Hagenston et al., 2008). Indeed, aged pyramidal neurons release more Ca^2+^ from intracellular stores than neurons from young adults after stimulation with an agonist of Group I mGluRs (McQuail et al., 2013). Therefore, impaired Group I mGluR function may reflect not only diminished contributions from mGluR5 that support PFC neural function and working memory via NMDARs, but also a relative strengthening of contributions from mGluR1 linked to intracellular Ca^2+^ signaling that disrupts working memory (Arnsten et al., 2012).

One prior study that assessed the effects of intra-PFC administration of mGluR ligands on working memory found that the mGluR2/3 agonist APDC dose-dependently impaired performance in rats performing a T-maze working memory task, whereas the mGluR2/3 antagonist LY341495 had no effect (Gregory et al., 2003). In contrast, the results of the current study showed that intra-PrL LY341495 impaired working memory accuracy, in the absence of effects on non-mnemonic aspects of task performance. The reasons for this apparent discrepancy between the two studies are unclear, although there were considerable methodological differences between the current study and that of Gregory et al. (2003). For example, the T-maze task employed by Gregory et al. (2003) likely engages medial temporal lobe mnemonic systems in addition to mPFC. Moreover, the target of mPFC infusions in that study may not have been restricted to the PrL subregion of mPFC as in the current report. Although additional studies in rats are needed to clarify the discrepancies between these two studies, it is notable that the current findings are in agreement with recent work by Jin et al. (2017), which assessed the effects of mGluR2/3 agonists in a nonhuman primate working memory task and in a rodent T-maze spatial working memory task. These authors reported that low doses of mGluR2/3 agonists enhanced both behavior (in primates and rodents) and PFC electrophysiological signatures of working memory (in primates; Jin et al., 2017). In the context of the current findings, blocking glutamate signaling via mGluR2/3 may impair working memory by altering regulation of extracellular glutamate levels, reducing modulation of ion channels in dendritic spines, or both. mGluR2/3 localizes to presynaptic terminals and glial processes where it regulates extracellular glutamate by inhibition of synaptic release (Tanabe et al., 1993; Muly et al., 2007) or stimulation of glutamate transporters on glial processes (Aronica et al., 2003; Corti et al., 2007). Indeed, presynaptic/glial mGluR2/3 is a prime target to counter dysregulated PFC glutamate signaling observed in schizophrenia (Patil et al., 2007; Moghaddam and Javitt, 2012; Vinson and Conn, 2012). Drugs that produce pathologically elevated release of glutamate and asynchronous PFC neural activity, including ketamine, PCP, and MK-801, are used widely to experimentally induce schizophrenia-like impairments in animal models, which are normalized by mGluR2/3 agonists (Moghaddam et al., 1997; Moghaddam and Adams, 1998; Lorrain et al., 2003; Jackson et al., 2004; Homayoun et al., 2005; Benneyworth et al., 2007; for review, see Maksymetz et al., 2017). Blocking mGluR2/3 recapitulates the excess extracellular glutamate produced by NMDAR antagonists which can, in turn, impair working memory (Dietrich et al., 2002; Xi et al., 2002). Parallel to regulation of extracellular glutamate are contributions from mGluR2/3 on dendritic spines that modulate PFC neural excitability via influences on postsynaptic ion channels. Activation of mGluR2/3 opposes cAMP-PKA signaling in PFC neurons that reduces persistent firing and impairs working memory (Ramos et al., 2006; Wang et al., 2007; for review, see Arnsten et al., 2005, 2012). A recent series of studies determined that inhibiting cAMP-PKA signaling through activation of postsynaptic mGluRs with either a mixed mGluR2/3 agonist or a selective mGluR3 agonist is sufficient to enhance persistent PFC neuronal firing during performance of a working memory task (Jin et al., 2017, 2018). Also relevant to actions in postsynaptic spines is the capacity of mGluR2/3 to potentiate NMDAR function. Stimulation of mGluR2/3 in dissociated mPFC pyramidal neurons potentiates NMDAR currents, especially in those NMDAR complexes that contain an NR2A subunit (Tyszkiewicz et al., 2004). The latter finding is highly consequential to working memory as previous work from our lab has determined that glutamate signaling via NR2A-NMDARs, and not NR2B-NMDARs, is essential for working memory (McQuail et al., 2016). Furthermore, loss of NR2A, but not NR2B, in the mPFC is correlated with severity of working memory impairment in aging (McQuail et al., 2016). When viewed together, these data suggest that loss of mGluR2/3, or possibly only mGluR3, can induce impaired regulation of glutamate signaling at both pre- and postsynaptic locations and, further, may exacerbate NMDAR-mediated deficits that arise with aging or in neuropsychiatric disease.

### Possible therapeutic benefits of targeting mGluRs 5 and 2/3

Selective blockade of mGluR5 or mGluR2/3 in young PrL recapitulates the age-related working memory impairments that are reliably observed across species (Oscar-Berman and Bonner, 1985; Dunnett et al., 1988; Rapp and Amaral, 1989; Bachevalier et al., 1991; Lamar and Resnick, 2004; Beas et al., 2013; Bañuelos et al., 2014; McQuail et al., 2016; Hernandez et al., 2017). In these studies, aged subjects perform comparably to their young counterparts at short delays but are disproportionately impaired relative to young as the delay over which they must maintain information increases. The loss of mGluR5 and mGluR2/3 from the aged mPFC and their necessity for working memory has important implications for the treatment of cognitive impairments that accompany normal aging. Contemporaneous with decline of mGluRs, loss of NMDARs in the PFC is also an established feature of normal aging linked to working memory decline (Piggott et al., 1992; Mitchell and Anderson, 1998; Bai et al., 2004; Magnusson et al., 2005; Das and Magnusson, 2008; McQuail et al., 2016). Given their functional interactions, we can speculate that age-related changes to ionotropic and mGluRs are interdependent features of dysregulated glutamate signaling contributing to age-related cognitive impairments. Strategies that target NMDARs yield, at best, moderate rescue of cognitive impairment in aged individuals (Baxter et al., 1994; Billard and Rouaud, 2007; Burgdorf et al., 2011; Panizzutti et al., 2014; McQuail et al., 2016). A shortcoming of NMDAR-directed treatments may be a failure to address concurrent age-related loss of mGluRs, which synergistically support glutamate signaling required for optimal working memory. Consequently, treatments that potentiate glutamate signaling via mGluR5 or mGluR2/3 separately or in concert with NMDAR-directed ligands are promising candidates to reverse age-related impairment of PFC-dependent cognition. Previous work showing positive effects of mGluR2/3 or mGluR3 agonists on PFC neural function and working memory, along with similar evidence from mGluR5 agonists, provides promising preliminary support for the notion that these receptors are viable therapeutic targets that can be leveraged to improve cognition (Ayala et al., 2009; Cleva and Olive, 2011; Jin et al., 2017, 2018). An important avenue of future research, however, will be determining whether modulation of mGluRs that enhances cognition in young adults can reverse cognitive impairments caused by changes to glutamate signaling in the aged brain.

## Synthesis

Reviewing Editor: Tim Bussey, University of Cambridge Decisions are customarily a result of the Reviewing Editor and the peer reviewers coming together and discussing their recommendations until a consensus is reached. When revisions are invited, a fact-based synthesis statement explaining their decision and outlining what is needed to prepare a revision will be listed below. The following reviewer(s) agreed to reveal their identity: Bernard Balleine, Martha Hvoslef-Eide. Note: If this manuscript was transferred from JNeurosci and a decision was made to accept the manuscript without peer review, a brief statement to this effect will instead be what is listed below.

It looked to me that you had done a very good job of addressing the reviewers' comments from JofN, and so I sent your revised MS back to just one of those reviewers, and one new reviewer. The JofN reviewer is satisfied with the revisions you made and has no further comments. The new reviewer raises a few relatively minor suggestions which I think would improve the MS. It seems to me the comment about multiple t-tests is one you would want to address. I would also draw your attention to the comment about the 2-lever DNMTP task and mediating responses, such that the task may no longer be measuring memory so much as the ability to employ such strategies (this has been shown explicitly to occur, e.g., Chudasama &Muir, 1997; Herremans et al., 1996). I realise you are aware of this problem and mention the possibility in your methods, but unfortunately nose-poking in the magazine may not help that much (for example have a look at Figure 1 in Chudasama and Muir to see a rat still ‘cheating’ in this situation). Nevertheless I think your findings in this study are valuable, as long as these caveats about the task are made clearly in the Discussion section. In addition, I have the following comments/queries:

1. In the results for the LY341495 experiment, the authors report a main effect of dose, as well as a dose by delay interaction. This is then followed by reporting that the vehicle vs dose comparison is significant for all three doses. I believe it is more appropriate when you have both significant a main effect and a significant main interaction to focus the post hoc assessment on the interaction, as the data is affected by two factors. I would like to see the results reported to reflect at which delay a specific dose was significant.

2. I see no mention of correcting for multiple comparisons, despite using several t-tests to assess significance between aged and young rats on several different target genes/proteins. Was this done? If so, please include in the manuscript. If not, please apply a multiple comparisons correction.

3. The authors do a nice job of putting the molecular and biochemical work into context, and the discussion of these results are thorough. The behavioural data receives less attention in the discussion, and would benefit from a more detailed comparison to previous work (e.g. expanding on how the methodological differences between the current work and Gregory et al. could account for the discrepancy of the two sets of findings).

4. It would also be nice to have a sentence added to the discussion commenting on how the impaired performance following antagonist infusions in the current study compares to the impaired performance the authors have previously observed in aged rats on the same task.

5. Was the administration of the doses counterbalanced between animals in e.g. a latin square design? If yes, please include in the manuscript. If not, please provide analysis to support the assumption that there are not order effects that can account for the behavioural differences observed.

6. Is there a reason that the control for the western blot (alpha-tubulin) is not shown?

7. The abstract had no mention of the reduction in mGluR4 or mGluR2. I appreciate that this was not consistent in both mRNA and protein levels, but I would prefer to see it mentioned nonetheless.
